# Analysis of morphological and quantitative changes in pathological myopia and perioperative changes in posterior scleral reinforcement using three-dimensional magnet resonance imaging

**DOI:** 10.3389/fbioe.2023.1242440

**Published:** 2023-12-15

**Authors:** Lin Liu, Hua Rong, Di Wu, He Xu, Qing He, Bei Du, Xuejun Zhang, Ruihua Wei

**Affiliations:** ^1^ Tianjin International Joint Research and Development Centre of Ophthalmology and Vision Science, Eye Institute and School of Optometry, Tianjin Medical University Eye Hospital, Tianjin, China; ^2^ Department of Radiology, Second Hospital of Tianjin Medical University, Tianjin, China

**Keywords:** high myopia, pathological myopia, 3D MRI, posterior staphyloma, posterior scleral reinforcement (PSR)

## Abstract

**Objective:** To compare the morphological and quantitative changes in pathological myopia (PM) and the perioperative changes in posterior scleral reinforcement (PSR) using three-dimensional magnetic resonance images (3D MRI).

**Methods:** A total of 49 patients with high myopia (HM; 98 eyes), 15 with pathological myopia (PM; 19 eyes), and 10 without high myopia (NORM; 20 eyes) were recruited between September 2019 and July 2021. The patients underwent measurements of refractive error and axial length, as well as 3D MRI of the eyeball. Python was used to analyze the 3D MRI images, calculate the vitreous volume, establish a topography of the height of the eyeball posterior surface, and calculate the rate of change in height (H). For the PM group undergoing PSR, changes in vitreous volume and the highest point of the eyeball posterior surface in four quadrants (temporal, subtemporal, nasal, and subnasal) were compared before and after PSR.

**Results:** The vitreous volume was smaller in the NORM group compared to the HM and PM groups (*p* < 0.01). The PM group had a larger volume than the HM group (*p* < 0.01). The H for the PM group was higher than that of the NORM and HM groups (*p* < 0.01). After PSR in the PM group, the total vitreous volume, as well as the volume in the subnasal and supratemporal quadrants, decreased (*p* < 0.05). Additionally, the highest point of the eyeball’s posterior surface was generally shifted to the upper nasal side. Finally, the shape and position of the scleral band after PSR were plotted.

**Conclusion:** 3D MRI is capable of a quantitative description of the eyeball morphology in PM and PSR. It allows for precise calculations of changes in vitreous volume and the H of the posterior surface. It also facilitates a meticulous analysis of the specific details of the scleral band following PSR.

## Highlights

• In clinical practice, it is crucial to evaluate eyeball morphology and posterior staphyloma comprehensively before posterior scleral reinforcement. But the routine ophthalmic clinical examination is difficult to achieve.
• 3D MRI provides detailed and overall information of eyeball images for pathological myopia and posterior staphyloma. It also can explore the changing details of eyeball morphology before and after posterior scleral reinforcement, and simulate the shape and relative position of scleral bands.
• 3D MRI might be an effective method for surgery plan before PSR and surgery evaluation after PSR in the future.

## 1 Introduction

The prevalence of high myopia (HM) is projected to increase significantly worldwide between 2000 and 2050. By 2050, it is estimated that 10% of the global population will have HM, representing a five-fold increase from 2% in 2000 (Holden et al., 2016). Within the HM population, the prevalence of pathological myopia (PM) is as high as 50%–70% (Ohno-Matsui et al., 2021). HM is characterized by a high degree of myopic refractive error, while PM is characterized by the presence of specific myopic lesions in the posterior fundus, such as myopic maculopathy, retinoschisis, choroidal neovascularization, chorioretinal atrophy, and posterior staphyloma (PS), which can lead to visual impairment and even blindness (Avila et al., 1984; Hayashi et al., 2010; Jong et al., 2021; Ohno-Matsui et al., 2021).

Currently, the assessment of eyeball and fundus morphology is commonly performed using ophthalmoscopy, B-mode ultrasound imaging, and optical coherence tomography (OCT). These methods primarily focus on detailed changes in the retina, choroid, and scleral structures in the posterior eyeball, rather than the overall shape. Studies have shown that even OCT images with a scanning range of 9 mm may not capture the entire extent of the posterior staphyloma in PM (Miyake et al., 2014; Asai et al., 2016; Kuo et al., 2016; Shinohara et al., 2016; Garcia-Ben et al., 2017; Ohno-Matsui and Jonas, 2019). Based on our clinical experience, we have observed that retinal folds after posterior scleral reinforcement (PSR) on OCT images make it challenging to appreciate the complete morphological changes of the retina.

In clinical research, *in vivo* magnetic resonance imaging (MRI) and planar measurements of different eye axis lines have been used to study eyeball morphology (Moriyama et al., 2012; Shinohara et al., 2013; Nagra et al., 2014; Wang et al., 2016). However, further investigation is needed to describe and clinically apply the three-dimensional (3D) MRI morphology of the eyeball (Ohno-Matsui, 2014; Yu et al., 2018). Computer processing of acquired 3D MRI images plays a crucial role in improving the efficiency and accuracy of MRI (Evans, 2016). The vitreous volume of the eyeball, which varies with axial length, can provide a direct and objective assessment of eyeball expansion. It serves as an important 3D indicator for overall observation of PM and PS.

The primary surgical treatment for PM is posterior scleral reinforcement (PSR) (Gerinec and Slezakova, 2001), where biological materials called scleral flaps are used to cover the thinner sclera in the posterior pole and contract and reinforce it. Over time, the scleral flap integrates into the weakened scleral area, exerting compressive force on the eyeball, which helps control axial elongation and inhibits the progression of high myopia (Xue et al., 2014; Shen et al., 2015; Zhu et al., 2016; Hu et al., 2018; Peng et al., 2019). PSR can also prevent retinal detachment and secondary retinoschisis associated with PM (Zhu et al., 2018). However, the success of the surgery depends on the positioning of the scleral band and the applied pressure. Otherwise, it can lead to severe complications such as retinal ciliary artery obstruction and visual field defects (Karabatsas et al., 1997; Wen et al., 2017).

In this study, we investigated the complete shape of the eyeball and adjacent structures using 3D MRI, which provides more detailed and accurate information compared to previous measurement techniques. We utilized Python to process and analyze the 3D MRI images of the eyeball to evaluate the morphological and quantitative changes in PM, as well as the perioperative changes in PSR, including vitreous volume and scleral band information.

## 2 Materials and methods

This prospective study was conducted at Tianjin Medical University Eye Hospital between September 2019 and July 2021, involving 73 participants selected through consecutive sampling. Exclusion criteria were applied, including individuals who had undergone scleral buckling, had a history of ocular trauma that could affect the shape of the eyeball, experienced claustrophobia, had a pacemaker or intraocular metal foreign body, or had systemic diseases. Informed consent was obtained from all participants, and for those under 16 years of age, consent was provided by their parents or guardians. The study adhered to the principles outlined in the Declaration of Helsinki and was approved by the ethics committee of Tianjin Medical University Eye Hospital (NO. 2020KY-04).

The participants were categorized into three groups based on the degree of myopic refractive error and MRI images (Jong et al., 2021). The NORM group included individuals with a spherical equivalent refractive error of both eyes ≥ −0.50 D. The HM group consisted of individuals with a spherical equivalent refractive error of both eyes ≤ −6.00 D but without posterior staphyloma (PS). The PM group comprised individuals with a spherical equivalent refractive error of both eyes ≤ −6.00 D and with PS. All participants underwent a comprehensive ophthalmic examination, which included assessments of best-corrected visual acuity (BCVA), slit-lamp and fundus examinations, and measurements of refractive error and intraocular pressure. The subjects in the PM group underwent posterior scleral reinforcement (PSR) as part of their treatment.

### 2.1 MR imaging

All eligible patients underwent a 3D MRI examination of the eye using the DiscoveryTM MR750 3.0T scanner (GE Healthcare, Milwaukee, WI, United States) equipped with an 8-channel head coil. Axial position images were obtained using a fast-recovery fast spin-echo acceleration sequence (3D FRFSE-XL) with the following imaging parameters: repetition time of 8,000 ms, echo time of 800 ms, field of view of 18 mm^2^ × 18 mm^2^, matrix size of 256 × 256, echo train length of 70, bandwidth of 62.5 Hz, slice thickness of 1 mm, and flip angle of 90°. The total scan time for each subject was 8 min and 41 s. T2-weighted images were used, allowing visualization of the outer surface of the intraocular fluid on the MRI. Consequently, the acquired images provided information on the vitreous volume in the posterior segment of the eye.

### 2.2 3D MRI postprocessing

We utilized Python 3.7.9 to process and analyze the MRI DICOM files. The Breadth-First Search algorithm was employed to extract the areas within the vitreous and cornea. Breadth-First Search (BFS) algorithm is a search algorithm used in graphs or tree data structures. It starts from a source node and explores nodes at the current level before moving on to nodes at the next level, and so on, until it reaches the target node or traverses the entire graph. In this study, it referred to starting from any point within the vitreous body and conducting a diffusion search in the surrounding area. Any point whose grayscale value differs within the threshold range from the previous point was included in the vitreous body matrix.

A Cartesian coordinate system was established with the *x* and *y*-axes parallel to the cross-section of the nuclear magnetic scan, with the origin at an arbitrary point. And then we calculated the geometric center coordinates of the vitreous body, denoted as v_c. Nextly, we translated the eye so that the geometric center of the vitreous body became the new origin, and subsequently calculated the coordinates of the geometric center of the cornea, denoted as c_c. This allowed us to obtain the vector v_c-c_c. We then used the Rodrigues’ formula to compute a vector that would rotate the vector v_c-c_c to align with the direction of the vector (0, 0, −1), which points vertically downward. With this vector, we constructed a rotation matrix for the entire eyeball, ensuring that the corneal direction of the eye was oriented vertically downward. This procedure ensured that all eyes were aligned in the same direction before proceeding with the subsequent calculation of the vitreous body volume.

**FIGURE 1:**
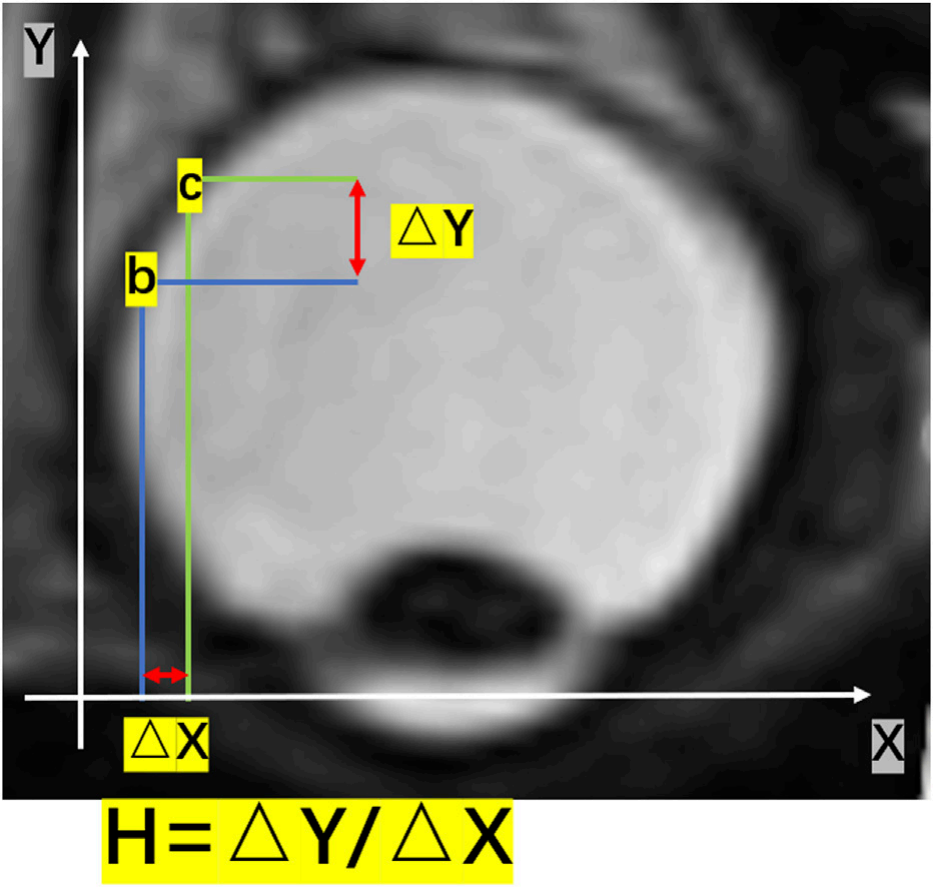
A schematic diagram of H in a two-dimensional image. The changing rate of the height of the posterior surface in the horizontal plane was given by H = ΔY/ΔX. ΔY represented the vertical change in height, and ΔX represented the change in distance in the horizontal direction.

We calculated the number of pixels within the vitreous area and converted it into volume. Furthermore, we determined the two points as b and c on the eyeball posterior surface. The point “b” and “c” were two randomed points on the eyeball posterior surface. As Figure 1 shown, It was an example of these two points. ΔX represented the horizontal distance between the two points b and c, and ΔY represented the vertical distance between the two points b and c. The changing rate of the height of the eyeball posterior surface in the horizontal plane was defined as H = ΔY/ΔX (Figure 1).

**FIGURE 2:**
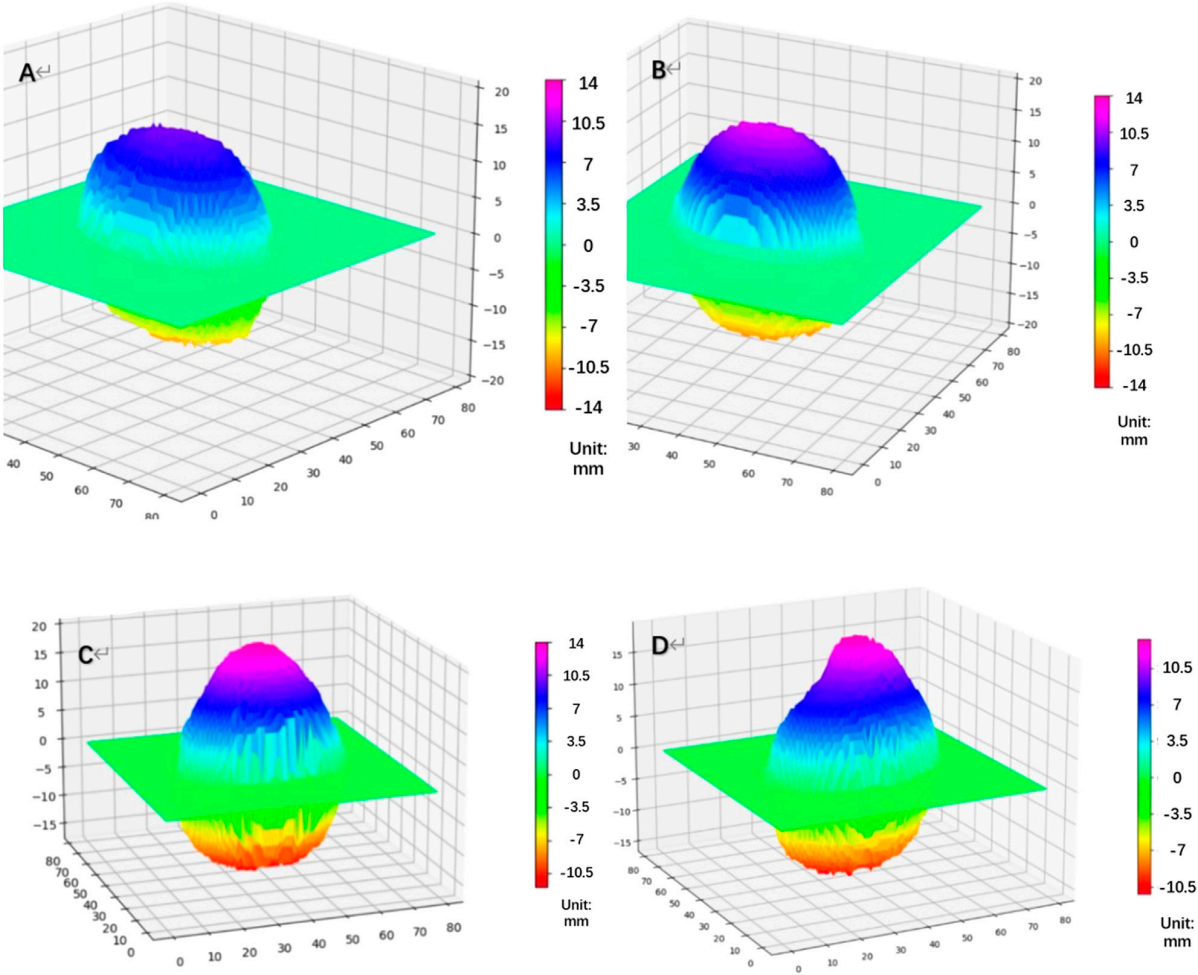
Topographic map of the height of the eyeball’s posterior surface [**(A)** NORM group, **(B)** HM group, **(C)** PM group: Pre-PSR, **(D)** PM group: Post-PSR] HM, high myopia; PM, pathological myopia; NORM, non-high myopia; PSR, posterior scleral reinforcement.

Based on the height value, we generated a topographic map of the posterior surface of the eyeball (Figure 2). Additionally, we calculated the changing rate of the height for each point on the eyeball posterior surface (H). For eyes undergoing posterior scleral reinforcement (PSR), we used the coronal plane that passing through the geometric center of the vitreous body to divide the eyeball into two parts. The posterior half was further divided into four quadrants using horizontal and sagittal planes: temporal, subtemporal, nasal, and subnasal regions. We individually calculated the volume of each part and analyzed the volume changes in each quadrant before and after surgery.

**FIGURE 3:**
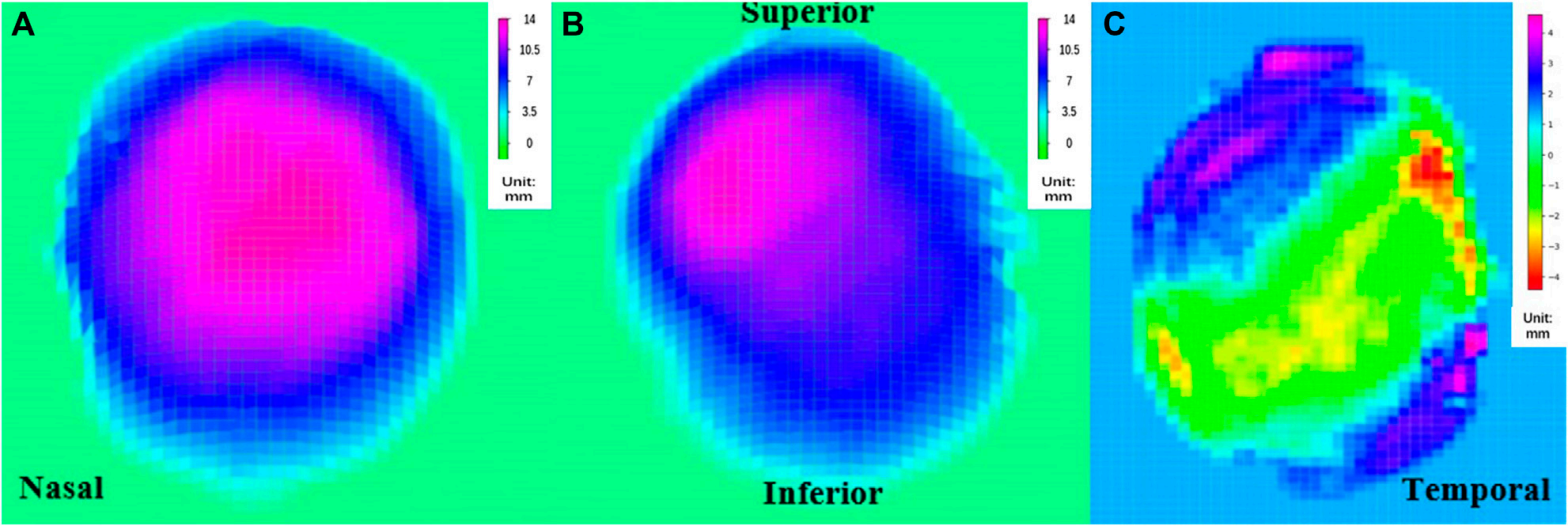
Top view of the rear surface height map **(A)** pre-PSR, **(B)** post-PSR, **(C)** the reconstructed image of the scleral band PSR, posterior scleral reinforcement.

By combining the volume changes, which provided information on coordinates, with a top-down view of the height map of the posterior surface (Figure 3A, B), we were able to compare the position of the highest point before and after PSR. The height matrices of the posterior surface before and after surgery were used to plot the differences in the height of the eyeball’s posterior surface, thereby visualizing the placement and extent of reinforcement of the scleral band (Figure 3C).

### 2.4 AL measurement

Axial length (AL) measurements were obtained using the Lenstar LS-900 (Haag-Streit AG, Berne, Switzerland). During the examination, the participants were instructed to keep their eyes open and focus on a target. To ensure accurate measurements and avoid errors, subjects were allowed to blink between measurements, ensuring an intact tear film. Data analysis was performed using the average of three repeated measurements, where the intrasession differences were no greater than 0.02 mm. This approach ensured the reliability and consistency of the AL measurements.

### 2.5 PSR procedure

All surgeries were performed by experienced doctors under a microscope to ensure precision and accuracy. Prior to the surgery, all patients provided their informed consent. The surgical strips used were made of homogeneous sclerae that underwent crosslinking and rigorous sterilization using 0.1% genipin, ensuring their safety and suitability for the procedure (Xue et al., 2014).

The surgical procedure was conducted under general anesthesia. Initially, the bulbar conjunctiva was incised at a 210° angle along the corneal limbus, with the inferior temporal aspect of the eye serving as the center point. Traction lines were created for the lateral rectus and inferior rectus muscles. The strips were then carefully inserted inwards and upwards, sequentially passing through the inferior oblique, lateral rectus, and inferior rectus muscles. Non-absorbable 5–0 sutures (Alcon) were used to secure the strips to the equatorial anterior sclera between the inferior and medial recti muscles. The lateral temporal end of the strips was anchored to the equatorial sclera between the superior and lateral recti muscles. Throughout the procedure, thorough checks were performed to ensure that the strips were correctly positioned and oriented before completing the surgery (Zhu et al., 2016).

This standardized surgical approach, along with the use of crosslinked scleral strips and meticulous intraoperative verification, aimed to ensure the effectiveness and success of the posterior scleral reinforcement procedure.

### 2.6 Statistical analysis

Statistical analyses were performed using SPSS statistical package 25 (SPSS, IBM, Chicago, IL, United States). The Kolmogorov-Smirnov test assessed the data’s normal distribution. Considering the inter-eye correlation between individuals, a linear mixed model was used to compared vitreous volumes and H among the NORM, HM, PM groups. Bonferroni *post hoc* analysis was used to compare differences between every two groups. The changes in vitreous volume and the volume in the four quadrants before and after PSR were further examined by Paired *t*-test. A *p*-values less than 0.05 was considered significantly.

## 3 Results

In total, 135 eyes of 73 patients who met the study inclusion criteria were analyzed. These included 19 eyes of 15 patients in the PM group, 96 eyes of 48 patients in the HM group and 20 eyes of 10 patients in NORM group. The age, spherical equivalence, axial length, and other parameters of the different groups were summarized in Table 1.

**TABLE 1:** Patients and initial datas of the eye examinations.

	PM	HM	NORM
No. Patients (eyes)	15 (19)	48 (96)	10 (20)
Men	6 (8)	22 (44)	4 (8)
Women	9 (11)	26 (52)	6 (12)
Age(y), mean ± SD	55 ± 19.4	49 ± 18.2	43 ± 12.0
SE(D), mean, mean ± SD	−13.5 ± 5.3	−8.22 ± 1.20	−0.14 ± 1.1
AL (mm), mean ± SD	30.03 ± 1.57	27.33 ± 0.47	23.46 ± 0.48
LogMAR, mean ± SD	0.74 ± 0.37	−0.01 ± 0.02	−0.03 ± 0.05

### 3.1 Vitreous volume

**FIGURE 4:**
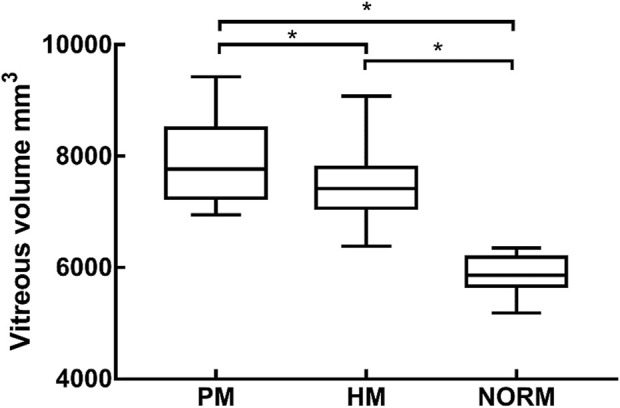
The vitreous volume of PM group, HM group, NORM group, * indicated a statistical difference between the two groups HM, high myopia; PM, pathological myopia; NORM, non-high myopia.

Univariate ANOVA revealed the following: the vitreous volume of the NORM group was 5,891 ± 1314 mm^3^, which was significantly smaller than that of the HM group at 7,426 ± 451 mm^3^ (*p* < 0.01). Meanwhile, the preoperative volume of the PM group was 7,896 ± 757 mm^3^, which was significantly larger than that of HM group (*p* < 0.01) (Table 2; Figure 4).

**TABLE 2:** The vitreous volume and H of NORM, HM and PM group.

Parameters	PM	HM	NORM	P (PM-HM)	P (PM-NOR)	P (HM-NOR)
Vitreous volume (mm^3^)	7896 ± 757	7426 ± 451	5891 ± 1314	<0.005^*^	<0.001^*^	<0.001^*^
H	4.22 ± 0.87	2.91 ± 0.26	2.89 ± 0.27	<0.001^*^	<0.001^*^	1.000

*Means significant differences between two groups using Bonferroni post hoc analysis.

### 3.2 Altitude change rate

**FIGURE 5:**
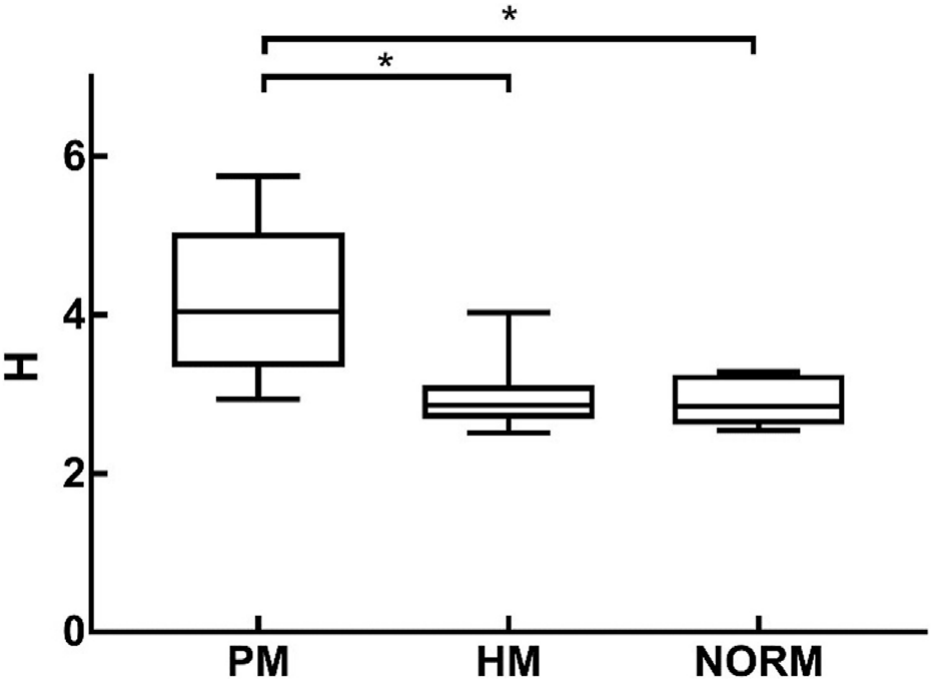
H of Pathological Myopia group (PM), High Myopia group (HM), NORM group (NORM), * indicated statistical differences between each of the two groups HM, high myopia; PM, pathological myopia; NORM, non-high myopia.

H was 2.89 ± 0.27 and 2.91 ± 0.26 in the NORM and HM groups, respectively. There was no significant difference in H between the two groups (*p* = 1.000). Preoperative H in the PM group was 4.22 ± 0.87, which was significantly higher than that in the NORM and HM groups (*p* < 0.01) (Figure 5).

### 3.3 Preoperative and postoperative volume changes

Generally, the postoperative vitreous volume in the PM group was 7,254 ± 906 mm^3^, indicating an average decrease of 642 mm compared with the preoperative volume (*p* < 0.05). By quadrants, first, the average volume of the upper nasal side of the posterior vitreous body increased by 84 mm^3^, although this difference was not significant (*p* = 0.177). Second, the volume of the inferior side of the nose was post-operatively significantly reduced by 104 mm^3^ (*p* < 0.05). Third, the volume of the supratemporal side was significantly reduced by 138 mm^3^ (*p* < 0.05). Lastly, the average volume of the inferior temporal side was reduced by 1 mm^3^, although this difference was not significant (*p* = 0.987) (Table 3).

**TABLE 3:** Changes in vitreous volume before and after PSR.

Location	Preoperative mm^3^	Postoperative mm^3^	Post- Pre mm^3^	P
Full vitreous	7896 ± 757	7254 ± 906	−642	<0.05^*^
Superior nasal	1020 ± 184	1105 ± 194	84	0.177
Inferior nasal	966 ± 145	862 ± 139	−104	<0.05^*^
Superior temporal	976 ± 148	837 ± 137	−138	<0.05^*^
Inferior temporal	910 ± 119	909 ± 175	−1	0.987

*Means significant differences using Paired t-test.

### 3.4 Changes in the position of the highest point of the posterior pole before and after PSR

**FIGURE 6:**
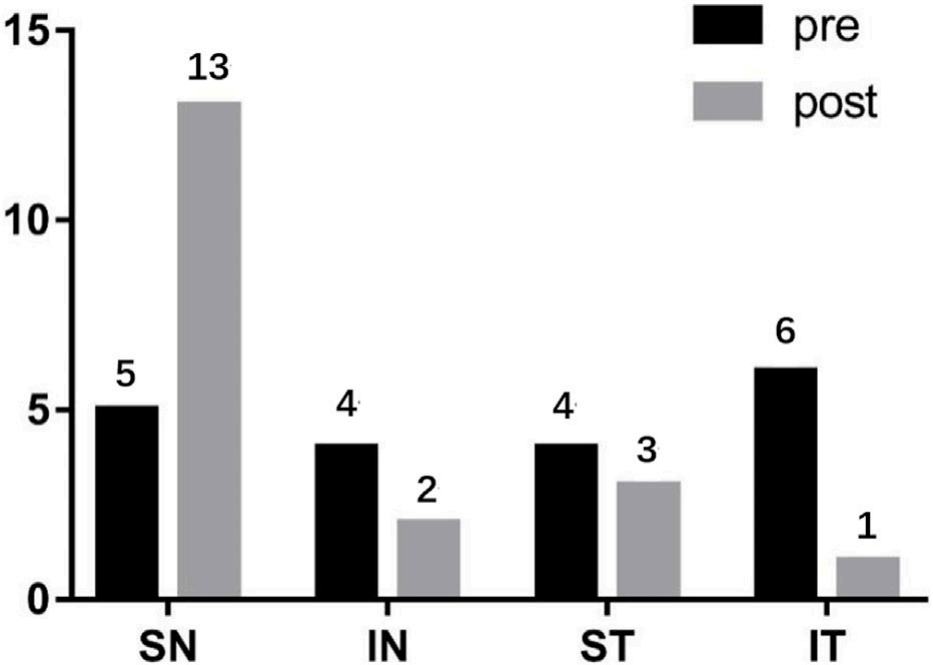
The changes of the highest point of the posterior pole before and after PSR SN: Superior nasal; IN: Inferior nasal; ST: Superior temporal; IT: Inferior temporal.

For the PM group, before PSR, the position of the highest point of the posterior pole was as follows: upper side of the nose, five eyes (26.3%); lower side of the nose, four eyes (21.1%); upper temporal side, four eyes (21.1%); and lower temporal side, six eyes (31.6%). After surgery, the position of the highest point of the posterior pole was as follows: upper side of the nose, 13 eyes (68.4%); lower side of the nose, two eyes (10.5%), upper temporal side, three eyes (15.8%); and lower temporal side, one eye (0.05%) (Figure 6).

## 4 Discussion

The complications arising from pathological myopia (PM) have a significant impact on visual impairment and blindness, particularly in East Asia. Posterior staphyloma (PS) is a distinct change in curvature at the posterior pole of the eyeball and serves as a reliable indicator of PM (Ohno-Matsui, 2014). Traditional methods of observing and analyzing eyeball morphology relied on one-dimensional or two-dimensional measurements, as well as subjective evaluations by clinicians (Jones and Luensmann, 2012; Tatewaki et al., 2019). General ophthalmic examinations may not fully visualize the shape of the staphyloma, further complicating the diagnosis and characterization of PS (Chae et al., 2011; Jo et al., 2012). Therefore, in our study, we employed Python 3.7.9 to process 3D MRI imaging, enabling us to obtain a comprehensive view of eyeball morphology. Volume renderings were utilized to reconstruct, analyze, and describe the data in a three-dimensional manner, presenting a novel approach for clinically studying eyeball morphology in PM.

In our study, we calculated the volume of the vitreous body and analyzed the differences among the NORM, HM, and PM groups. Previous research has indicated a positive correlation between vitreous volume and axial length, as an increase in axial length is primarily attributed to an expansion of the vitreous cavity (Nagra et al., 2014). Similarly, we found that the vitreous volume was larger in the HM and PM groups compared to the NORM group.

To delve deeper into the morphology of the eyeball, we calculated the height (H) and analyzed its variations among the NORM, HM, and PM groups. Our findings revealed that the H of the PM group was significantly greater than that of the NORM and HM groups. The elevated H was attributed to a sudden change in height at the edge of the PS. Interestingly, we observed that the absence of PS, sudden changes in the posterior surface, and a lack of significant H increase were characteristics found in eyes without PM-associated axial elongation. In PM, axial elongation leads to thinning of the posterior pole sclera, reduced biomechanical properties, the development of PS, and retinoschisis. Consequently, patients experience impaired corrected vision and, in severe cases, even blindness, significantly affecting their quality of life (Jones and Luensmann, 2012). posterior scleral reinforcement (PSR) can be employed clinically to strengthen the mechanical integrity of the posterior sclera, control axial elongation, and reattach a detached retina (Shen et al., 2015). However, PSR is associated with potential complications, including local ocular compression protrusion, choroidal atrophy, choroidal neovascularization, post-ciliary retinal artery occlusion, visual field defects, and capillary damage (Wen et al., 2017; Cao et al., 2020). Wen et al. suggested that the shape and positional stability of the allogeneic scleral reinforcement strip are crucial factors affecting the postoperative efficacy of PSR (Wen et al., 2017). Hence, it is imperative to assess the details of the scleral band postoperatively to evaluate the surgical outcomes.

### 4.1 Perioperative changes in posterior scleral reinforcement

In this study, we conducted a comparative analysis of 3D MRI images before and after posterior scleral reinforcement (PSR) using allogeneic scleral bands. By observing the indentations created by the bands on the sclera, we were able to assess changes in the volume of the vitreous body, height, and position of the posterior pole’s highest point.

Our findings revealed a decrease in vitreous volume after surgery, which can be attributed to the compression exerted by the reinforcing bands on the sclera. Specifically, the mean volume of the upper nasal side showed a slight increase of 342 pixels postoperatively compared to the preoperative measurements, although this difference was not statistically significant. On the other hand, the volume of the upper temporal and subnasal sides exhibited significant reductions, while the volume of the subtemporal side did not show a significant change. These volume changes can be explained by the positioning of the reinforcement bands, which passed through the temporal to lower rectus external muscles below the optic nerve and under the inferior rectus muscle. As a result, the upper nasal side appeared to be more prominent, leading to a concentration of the posterior pole’s highest point on this side (68.4%) after surgery.

The observed patterns of changes in the distribution of the posterior eyeball’s volume among the four quadrants were consistent with the alterations in vitreous volume and the position of the posterior surface’s highest point. Thus, 3D MRI imaging provided valuable insights into the shape and position of the allogeneic scleral reinforcement bands.

Overall, this study demonstrated the utility of 3D MRI imaging for evaluating changes in the posterior eyeball’s morphology following PSR and provided valuable information on the effects of scleral band placement on vitreous volume, distribution of the highest point, and changes in the quadrants of the posterior surface.

## 5 Limitations

This article has a few limitations that should be considered. Firstly, the sample size of patients who underwent posterior scleral reinforcement (PSR) was small. This limited sample size may affect the generalizability of the findings. Additionally, compared to other studies, our investigation did not observe any complications or band deviations. It is important to acknowledge that the absence of complications may not be representative of the overall experience with PSR. Furthermore, future studies could benefit from focusing on the localization of extraocular muscles, optic nerves, and fossa veins. By accurately identifying and quantifying the anatomical structures involved, quantitative surgical designs could be developed to enhance the precision and efficacy of PSR procedures.

## 6 Conclusion

The utilization of computer processing to analyze 3D MRI images of the eyeball offers valuable insights into the topographic height map of the posterior surface and allows for the calculation of vitreous volume. However, the most significant finding of this study lies in the determination of the shape and position of the scleral band following posterior scleral reinforcement (PSR). This technique has the potential to be further developed, enabling 3D MRI images of the eyeball to provide enhanced technical support for surgical design and prognostic evaluation in PSR.

## Data Availability

The raw data supporting the conclusion of this article will be made available by the authors, without undue reservation.
